# Developing Computer Vision Models for Classifying Grain Shapes of Crushed Stone

**DOI:** 10.3390/s25061914

**Published:** 2025-03-19

**Authors:** Alexey N. Beskopylny, Evgenii M. Shcherban’, Sergey A. Stel’makh, Alexandr A. Shilov, Irina Razveeva, Diana Elshaeva, Andrei Chernil’nik, Gleb Onore

**Affiliations:** 1 Department of Transport Systems, Faculty of Roads and Transport Systems, Don State Technical University, 344003 Rostov-on-Don, Russia; 2 Department of Engineering Geometry and Computer Graphics, Don State Technical University, 344003 Rostov-on-Don, Russia; au-geen@mail.ru; 3 Department of Unique Buildings and Constructions Engineering, Don State Technical University, 344003 Rostov-on-Don, Russia; sergej.stelmax@mail.ru (S.A.S.); alexandr_shilov@inbox.ru (A.A.S.); razveevai@mail.ru (I.R.); diana.elshaeva@yandex.ru (D.E.); chernila_a@mail.ru (A.C.); 4 Institute of Applied Computer Science, University ITMO, Kronverksky Pr. 49, 197101 Saint Petersburg, Russia; onoreg@mail.ru

**Keywords:** crushed stone, flakiness index, plate-like grains, needle-like grains, computer vision, neural network, point cloud processing

## Abstract

In the construction industry, along with traditional approaches for the visual and instrumental assessment of building materials, methods based on intelligent algorithms are increasingly appearing; in particular, machine learning and neural network technologies. The utilization of modern technologies enables us to enhance building processes to a new quality level, decreasing the construction pace without precision losses compared to traditional methods. This research introduces a novel method for characterizing crushed stone grain morphology using the application of specially designed three-dimensional computer vision neural networks to point data clouds. Flakiness affects the strength, adhesion, and location of crushed stone grains. So, calculating this indicator by determining the planar dimensions of each particle in the crushed stone is necessary for the assessment of its suitability for various types of construction work. Architectures based on PointNet and PointCloudTransformer are chosen as the basis for the classification algorithms. The input data were 3D images of crushed stone grains, the shapes of which were divided into needle-shaped, plate-shaped, and cubic classes. The accuracy quality metric achieved during the training of both models was 0.86. Using intelligent algorithms, along with grain analysis methods via manual selection, sieve analysis, or using special equipment, will reduce manual labor and can also serve as an additional source for verifying the quality of building materials at various stages of construction.

## 1. Introduction

New products in the field of artificial intelligence (AI) are increasingly attracting interest in all areas of human activity, and the construction sector is no exception [1,2]. By simulating human cognitive functions, intelligent algorithms act as additional tools for analyzing and evaluating processes at all stages of the life cycle of construction projects. AI is becoming an important tool on the path to automation and digitalization in this sector of the economy [3,4,5]. When performing specific tasks in the construction sector, AI demonstrates results comparable to or even superior to the results of human intellectual activity [6,7,8]. The most common technologies today in the subject area under consideration are computer vision and intelligent decision support. Considering in more detail practical examples of intellectualization, it is worth highlighting the optimization of design solutions through the analysis of big data in the construction sector [9,10,11,12]. Analysis of the internal and external characteristics of already completed projects allows us to identify positive and negative factors in their development, as well as potential risks. Big data is used most successfully in international projects [13,14]. The volume of these data is projected to grow exponentially due to the proliferation of technologies, including sensor networks and the Internet of Things [15]. Future key areas include big data research in the field of construction safety, site management, heritage conservation, waste minimization, and quality improvement [16]. Incorporating modern best practices in decision-making within the construction sector is subject to ongoing annual guideline revisions [17,18]. Intelligent technologies allow for the planning and monitoring of projects of all sizes, as well as predicting possible risks and delays [19,20,21]. Machine learning algorithms are becoming a part of various types of monitoring systems at construction sites [22,23,24], and also help in detecting defects and finding deviations from norms and standards [25,26,27]. It is known that neural networks are trained using data obtained from finite element analysis under static loads and dynamic excitations for monitoring the condition of bridges [28]. The machine learning model allows for predicting the displacement of high-rise structures under vertical and lateral loads with an accuracy of over 99% [29]. The QD-LUBE method demonstrates good efficiency for assessing the seismic characteristics of a building [30]. Convolutional neural networks show good efficiency in monitoring the condition of aluminum building structures [31]. Many studies propose various machine learning methods to monitor the condition of building objects, structures, products, and materials and to assess and classify the recorded defects [32,33,34].

A detailed examination of the intelligent technologies’ applications for building materials, products, and structures reveals several of the most common task classes, such as segmentation [35,36], detection [37,38], and classification [39,40]. Several works [41,42,43] are devoted to the analysis of bulk stone materials, which consider various classification problems depending on the color, morphology, and textural characteristics. An analysis of the scientific publications showed the interest of researchers in implementing intelligent models for the analysis of building materials, in particular, bulk stone [13,44]. Nevertheless, there is a shortage of studies in the literature considering the application of neural networks for the analysis of the grain shape of bulk stone materials and the optimization of the quality control process for the parameter “content of plate-shaped (flaky) and needle-shaped grains”. Currently, crushed stone is one of the key building materials, which is used in huge quantities in the construction of linear objects, buildings, and structures for industrial and civil purposes [13,41,42,43,44,45,46]. In the production of crushed stone, quality control is conducted by conducting laboratory tests according to approved regulatory methods, which require large labor and time resources. Hence, there is a need to improve the quality control system and increase its efficiency. Rapid detection of the content of plate-like and needle-shaped grains using AI will significantly improve the quality of finished products and reduce time and labor resources during factory quality control. In this regard, the scientific novelty of this study comprises creating a database of 3D images of crushed stone, as well as developing an alternative approach to determining the content of plate-like and needle-shaped grains.

The aim of the study is to develop computer vision models for identifying the shape of crushed stone grains. The hypothesis of the study is as follows: neural networks can be used to classify crushed stone grains by analyzing their geometric parameters. The objectives of this study include the following:

→ Collecting an empirical database of crushed stone grain images;
→ Carrying out the augmentation process to expand the set of representative data;
→ Selecting neural network architectures that are most suitable for solving the tasks;
→ Training the selected algorithms;
→ Testing the trained algorithms on a test sample;
→ Analysis of the obtained results according to the main quality metrics of classification models.

The theoretical significance of the study lies in expanding the understanding of the possibilities of using artificial intelligence technologies, in particular neural networks, to analyze the grains of stone building material based on their morphological characteristics. The practical significance of the work lies in developing an algorithm for the intelligent analysis of 3D images of crushed stone grains of various classes, which can serve as an additional source for verifying the quality of the material at various stages of construction.

Additionally, it is necessary to highlight the existing alternative methods, potential biases, and industry applications related to the use of artificial intelligence or its absence in specific types of work. In this case, the testing of crushed stone grains is a manual, painstaking work that requires significant human resources, time, and labor costs and does not provide the required accuracy. At the same time, the use of artificial intelligence to solve these problems will save financial and human resources. A similar approach can be characterized, for example, by work in laboratories related to wheat grains or food products, as well as in clothing production, where defects noticeable to the human eye can be classified using computer vision. Thus, the proposed approach can be projected to other industries with the potential to save a significant amount of financial and human resources.

## 2. Materials and Methods

### 2.1. Grain Shape of Crushed Stone and Methods for Determining It

**Figure 1:**
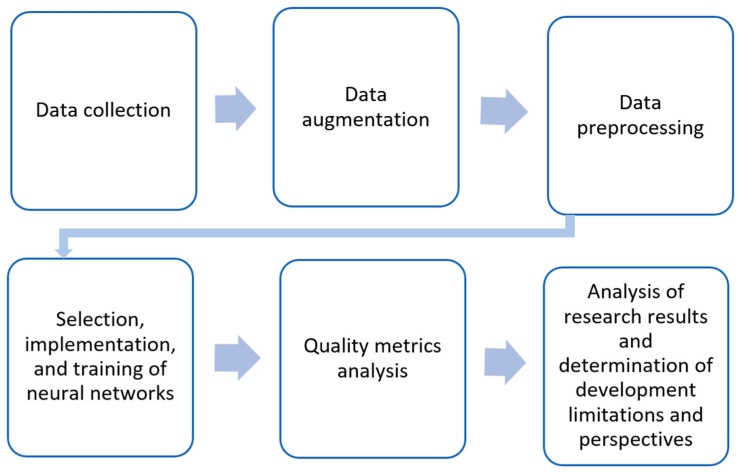
Study design.

Figure 1 shows the general scheme of this study, divided into 6 stages.

The material selected for analysis was crushed stone (Pavlovsk Nerud, Pavlovsk, Russia—geographic coordinates: 50.416284, 40.169466), the grain shapes of which can be divided into three classes: acicular, plate-shaped, and cubic. A grain is considered plate-shaped if its thickness is three or more times less than its length and acicular if its length is three or more times greater than its width. Most cuboidal grains exhibit polyhedral morphology. As a rule, the thickness, width, and length of such grains should be approximately equivalent.

The quality indicator of crushed stone, “content of grains of plate-shaped (flaky) and acicular shapes”, was determined in accordance with the requirements of the methodology [47]. It presents two main methods for determining the percentage of grain content of flaky and acicular shapes.

**Figure 2:**
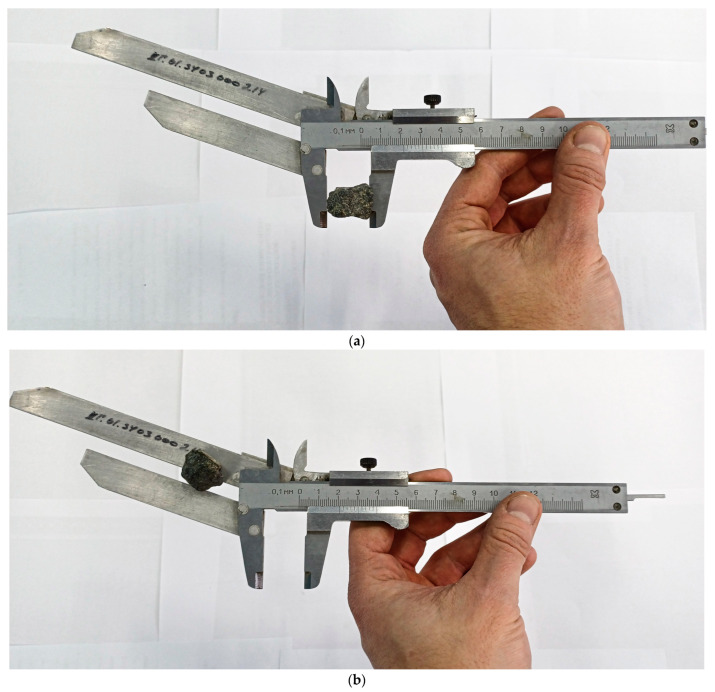
Determination of the content of plate-shaped (flaky) and needle-shaped grains using a template model 221111 (Dorstroypribor, Moscow, Russia): (**a**) fixing the largest grain size between the jaws; (**b**) passing the grain of the smallest size between the template plates.

The first method involves visually sorting the crushed stone grains. The testing procedure for the first method includes the following stages:

→ The preparation of a laboratory sample of crushed stone of one grain fraction;
→ Weighing the sample and selecting from it grains of a lamellar and needle-shaped form;
→ Determining the ratio of grain sizes using a movable template: First, the grain to be measured is placed with its largest size between the jaws, and its position is fixed (Figure 2a); then, the grain is passed with its smallest size between the plates of the template and installed at a distance three times smaller (Figure 2b). If the grain passes between the jaws, then it is classified as a grain of lamellar or needle-shaped form.
→ The percentage ratio of grains of a lamellar and needle-shaped form is determined by the ratio of the mass of grains of a lamellar and needle-shaped form to the mass of the original sample.

The second method involves sifting the crushed stone sample through special slotted sieves. The procedure for conducting the test according to the second method includes the following stages:

– Preparing a crushed stone sample of one fraction and weighing it;
– Sifting the sample through slotted sieves;
– Weighing the crushed stone grains that have passed through the slotted sieves.

The percentage content of plate-shaped and needle-shaped grains is also determined by the ratio of the mass of the grains that have passed through the sieve to the mass of the original sample.

### 2.2. Data Collection, Annotation, and Augmentation

**Figure 3:**
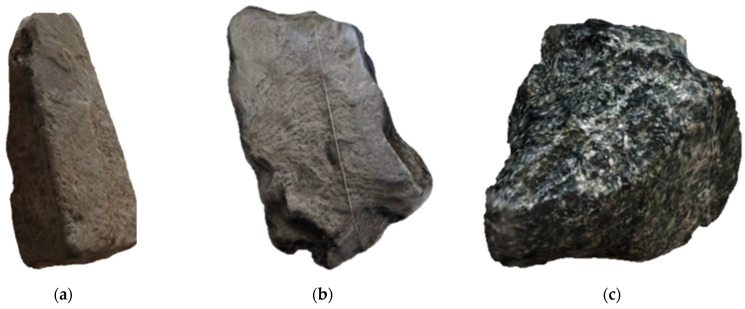
Crushed stone grain classes: (**a**) acicular, (**b**) plate-shaped, and (**c**) cuboid.

To create the empirical base of the study, 45 samples of crushed stone were selected in laboratory conditions, including 15 class needle-shaped (Figure 3a), 15 class plate-shaped (Figure 3b), and 15 class cuboids (Figure 3c). They were photographed in the .usdz (augmented reality file format) format using a smartphone (iPhone 15) with a camera resolution of 48 MP (Apple, Cupertino, CA, USA). After that, the data were converted to .obj (a 3D graphics file format used to store 3D geometry: vertices, faces, texture coordinates, and normals) using the Trimesh library (https://github.com/mikedh/trimesh) (accessed on 15 March 2025) for reading, writing, and manipulating 3D triangular faces (version 4.6.1).

**Figure 4:**
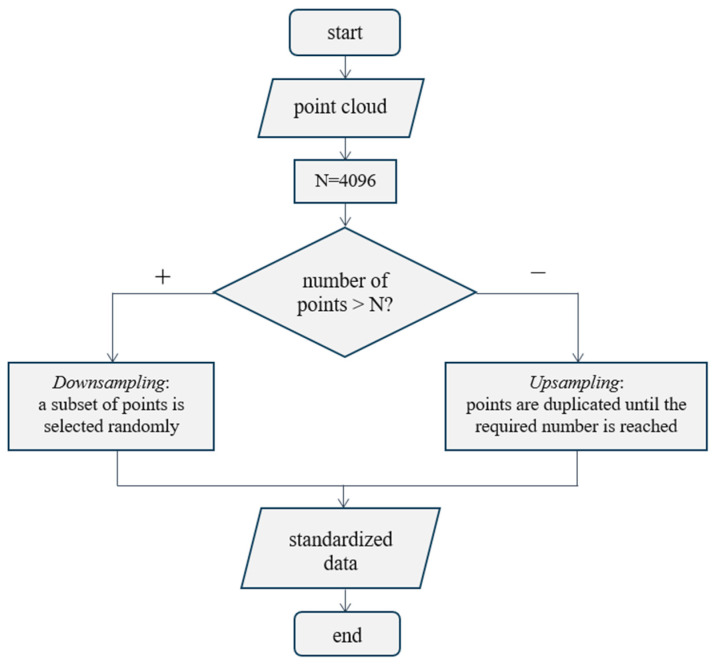
Resampling process.

The .obj format is a widely used format for storing 3D models, which contain information about vertices (points), edges, and other geometric data. During the loading process, the model is converted into a data structure that allows for the easy manipulation of vertices and edges. After loading the crushed stone model, vertices are extracted, which are a set of points in a three-dimensional space. These vertices are the basis for further analysis. Each vertex is described by three coordinates (x, y, z), which determine its position in space. Thus, the set of vertices is a point cloud describing the shape of the 3D model. To ensure data uniformity and simplify subsequent processing, the number of points in the cloud is reduced to a fixed size. The source data covers a wide range of point cloud sizes, from 3000 to 12,000. The study empirically verified that processing a large number of values significantly increases computational costs and training time but does not lead to a significant increase in model accuracy. To ensure a balanced data representation, it was decided to use 4096 points, which corresponds to a dimension multiple of 1024. This choice allows for the maintenance of sufficient object detail with an optimal ratio of computational costs and classification quality when using the neural network architectures planned for implementation. Data standardization is extremely important for deep learning tasks since the input data must have the same dimension. To reduce the number of points in three-dimensional space to a single number for each object, a resampling process is used, which includes two main approaches shown in the diagram (Figure 4).

The input is a point cloud, the size of which can be arbitrary. If the number of points in the cloud exceeds the target value *N* = 4096, then a subset of points is randomly selected (downsampling); otherwise, they are duplicated until the required number is reached (upsampling), which is represented by the “+” branch and the “−” branch in the branching block of the algorithm. At the output of the conditional block, a standardized point cloud is obtained.

**Figure 5:**
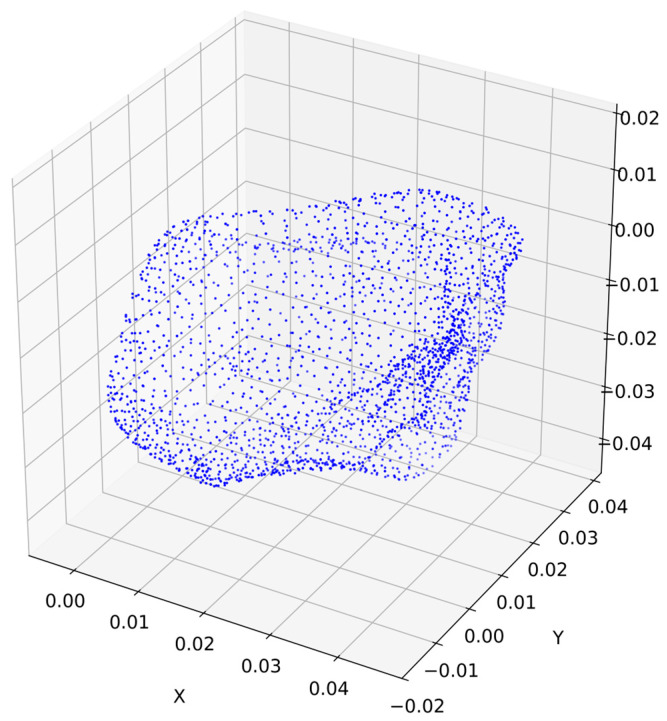
Standardized point cloud.

Figure 5 shows a point cloud standardized to a given value; the dots represent a cloud in three-dimensional space XYZ (the axes of the figure).

The visualized cloud confirms that the selected quantity *N* = 4096 allows for the preservation of all the main morphological characteristics of the grain.

To improve the quality and diversity of the data set, a comprehensive approach to augmenting the data used to classify 3D objects (grains) was implemented. Both general (basic) transformations and specific modifications adapted to the feature of the needle-like class were applied to the objects saved in 3D models in the .obj format.

Regardless of the grain class, the following basic transformations were applied to all objects:

1. Random rotation. The object is rotated around all three axes (X, Y, Z) by random angles in the range from −π to π. This allows the model to be invariant with the orientation of the object in space. Implemented using the Euler rotation matrix [48].
2. Random scaling. The object is scaled along all three axes by a random factor in the range from 0.5 to 1.5. This allows the model to be robust to changes in the object’s size. The value of the random transformation coefficient is determined randomly within certain limits that are possible within each class.
3. Adding noise. Gaussian noise with a zero mean and a standard deviation of 0.01 is added to the object’s vertices [49]. This helps the model be robust to small distortions in the data.
4. Reflection. The object is reflected along one of the three axes (X, Y, or Z) with a 50% probability. This increases the diversity of the data and helps the model account for the symmetrical properties of objects.

Additional augmentations are applied to needle-like objects that take into account their elongated shape:

1. Scaling along the main axis.

The main axis of the object is determined (the axis of the largest size). The object is scaled along this axis by a factor of 3.5 to 5.0, which enhances its elongated shape. Scaling by a factor of 0.4 to 0.8 is applied to the remaining axes to preserve the proportions. These transformations allow the model to better recognize needle-like objects, even if their shape varies. The values of these transformation coefficients are determined randomly within certain limits possible within each class.

The original models are classified by geometric features. The original instances are moved to the test set for a more relevant (correct) assessment. For each original instance, a set of augmented images is generated using the random transformations described above. The augmented objects are distributed between the training and test sets until the target number is reached (for the test set—50, for the training set—250). The test and training set sizes are ~80/20 (exactly 83/17), which is a popular value due to the balance between model training and evaluation.

The use of standardization and augmentation of this kind has a positive effect on the quality of machine learning models working with three-dimensional data for the following reasons.

1. Data unification. Resampling of points ensures the same size of input data, which simplifies the model architecture and the training process.
2. Increasing data diversity. Thanks to the selected set of modifications that are correct for the objects under consideration, a more diverse data set appears, which improves the generalizing ability of the model. It is important to note that, for needle-shaped grains, an algorithm was created that implements specific augmentations that take into account the unique characteristics of objects of this class, which increase the accuracy and reliability of classifiers.

### 2.3. Selection of Neural Network Architectures

Since the data set prepared for analysis is a cloud of points, it is advisable to select neural networks for their processing that are specially designed to work with three-dimensional data presented in the form of point clouds. In this study, models based on the PointNet [50] and PointCloudTransformer [51] architectures are used. The PointNet model is designed to process point clouds and uses convolutional and fully connected layers for feature extraction. The main components are as follows.

1. Input Transform Network: a convolutional network that transforms input 3D coordinates into a feature space.
2. Feature Transform Network: an additional convolutional network for further feature extraction.
3. Global Feature Aggregation: max pooling is used to aggregate features across all points.
4. Classifier: a multilayer perceptron that predicts the class of an object.

The architecture used has a number of differences from the original PointNet implementation [50]:

– There are no matrix transformations (alignment networks);
– Local features are excluded;
– Only max pooling is used, without additional operations with local features.

Due to these differences, a number of advantages are achieved, namely simplicity and efficiency due to a reduction in the number of parameters, as well as good scalability and low computational costs. At the same time, the neural network in this implementation is less resistant to noise and rotation than the original PointNet.

**Figure 6:**
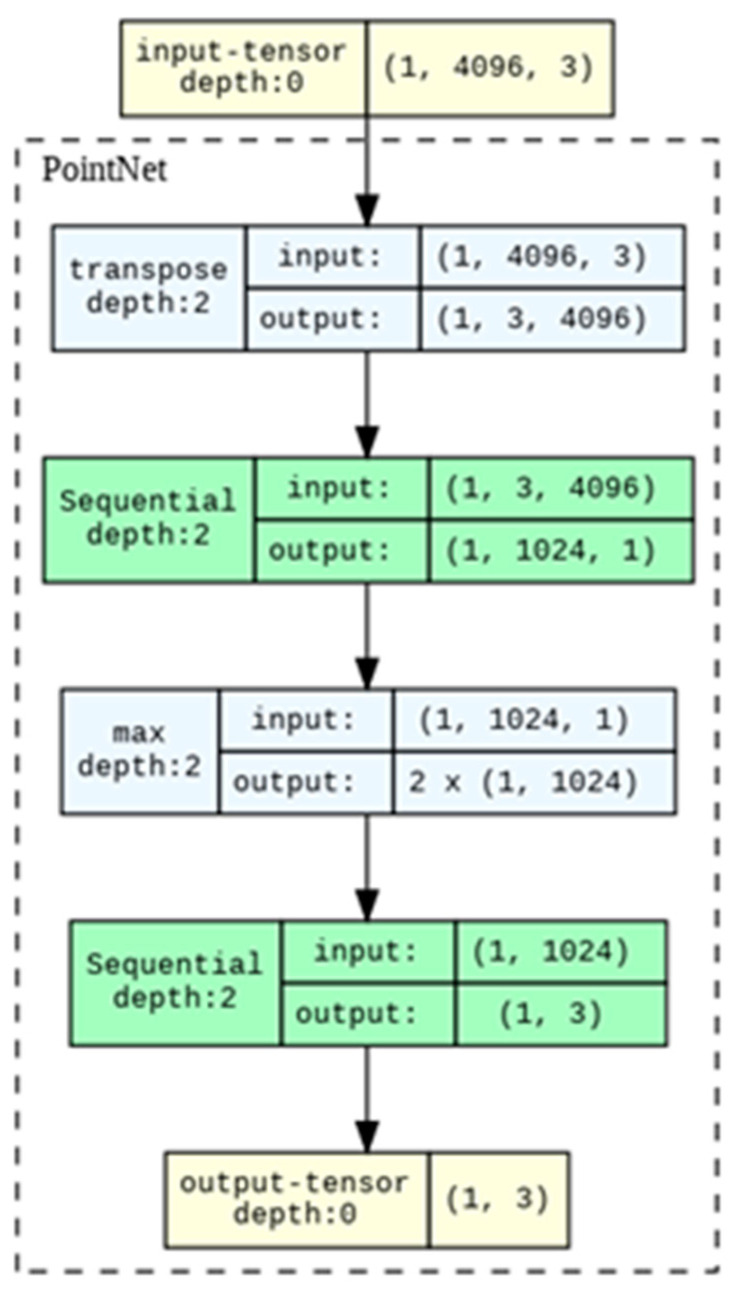
PointNet-based architecture.

Figure 6 shows a shortened PointNet diagram; the input is a tensor of size (1, 4096, 3), where 1 is the batch size, 4096 is the number of points in the cloud, and 3 is the number of coordinates for each point; the output is (1, 3), where 1 is the batch size and 3 is the number of classes in the model. The output values are logits, which are then transformed into probabilities using Softmax to obtain the final result.

**Figure 7:**
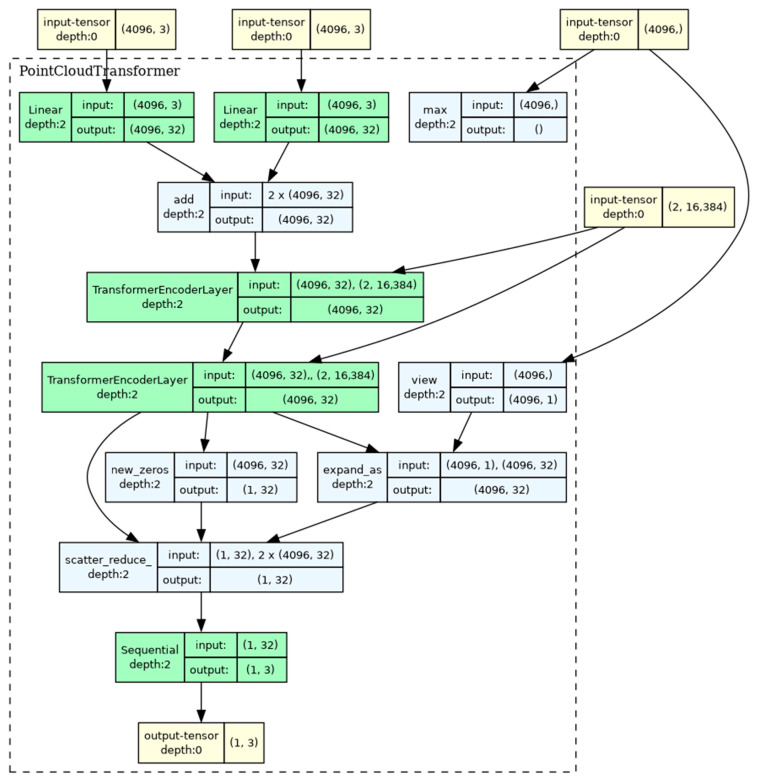
PointCloudTransformer-based architecture.

The second model chosen for implementation is represented by the PointCloudTransformer architecture (Figure 7) [51].

This model is based on the self-attention mechanism and is also designed to process point clouds. The key elements are the following.

1. Linear projections (input projection and positional encoding) for transforming input 3D coordinates.
2. TransformerEncoderLayer.
3. Global max pooling for combining information from the entire point cloud.
4. A classifier represented by a multilayer perceptron with normalization and dropout for class prediction.

Thanks to the self-attention mechanism, this network captures global dependencies between points. At the same time, high computational complexity O(N²) is observed for a large number of points.

## 3. Results and Discussion

### 3.1. PointNet-Based Model Training

Table 1 shows the parameters set at the beginning of PointNet-based neural network training. It is worth noting that FocalLoss (1) was used as the loss function. The learning rate was 0.001, and the number of epochs was 50.

(1) $$FL\left(p_{t}\right)=-\alpha_{t}{\left(1-p_{t}\right)}^{\gamma }\log\left(p_{t}\right)$$

$p_{t}$—probability of belonging to the target class;

$\alpha_{t}$—weighting factor for class balance;

*γ*—a modulating parameter that increases the importance of difficult examples.

**Figure 8:**
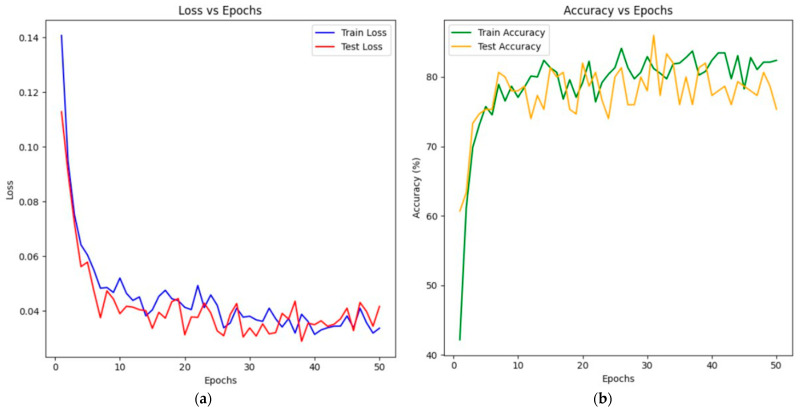
Training a model based on PointNet: (**a**) Loss vs. Epoch plot; (**b**) Accuracy vs. Epoch plot.

Figure 8a shows the model training process, and Figure 8b shows the growth of the Accuracy metric during this process.

In both cases, the epoch values are plotted along the X-axis, and the loss function changes for the first figure and the quality metric for the second are plotted along the OY-axis. According to the figure, a decrease in the model error is observed as the number of epochs increases, while the Accuracy metric increases both on the training and test samples.

**Figure 9:**
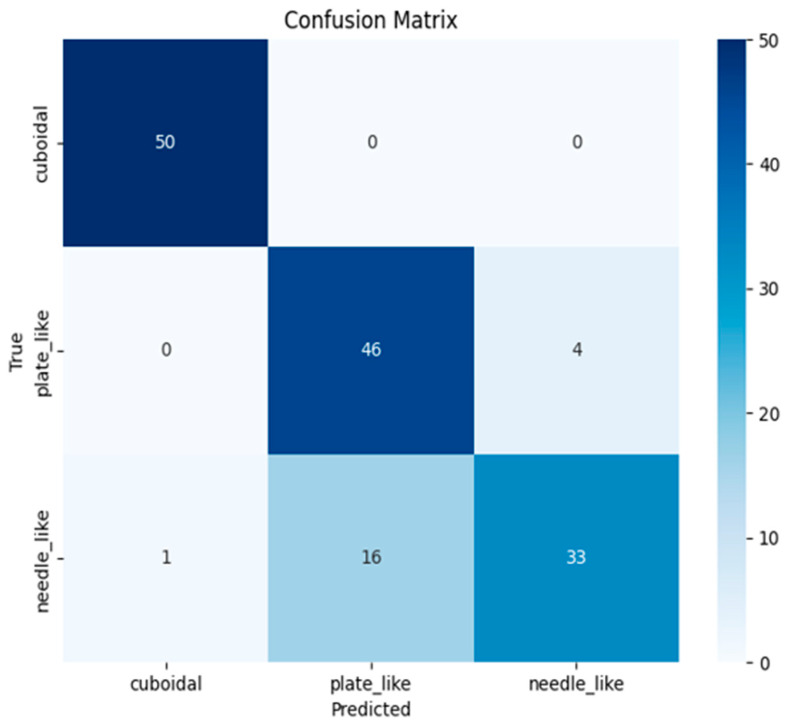
Classification error matrix for PointNet.

To assess the quality of multi-class classification, the error matrix shown in Figure 9 is visualized. The matrix is a table consisting of three rows and three columns, which corresponds to the number of classes, with the rows corresponding to the actual classes and the columns corresponding to the predicted ones. The cells of the matrix contain the numbers of the examples classified accordingly. The numbers in the cells at the intersection of rows and columns for classes of the same name (when the predicted class corresponds to the actual one) determine the number of correctly classified examples. Matrix cells containing zero values indicate the absence of model errors in the corresponding combinations of actual and predicted classes, which means that the model is able to identify differences between these types of crushed stone grains with maximum accuracy. For example, the network distinguishes between cuboidal and plate_like classes, as well as cuboidal and needle_like. In turn, the needle_like class has the minimum number of correctly classified examples. The network most often confuses this class with plate_like, which is due to the visual similarity of these classes. A strategy to reduce confusion seems to be to increase the data set size.

For a comprehensive assessment of the quality of the model, metrics such as *Precision*, *Recall*, and the *F*1 were calculated, determined by the following formulas:

(2) $$Precision=\frac{TP}{TP+FP}$$

(3) $$Recall=\frac{TP}{TP+FN}$$

(4) $$F1=\frac{2\times Precision\times Recall}{Precision+Recall}$$

Precision shows what proportion of predicted positive classes are actually positive. Recall measures what proportion of real positive examples were correctly detected by the model. The *F*1-score is the harmonic mean of *Precision* and *Recall*, balancing their ratio. The obtained values for *Precision*, *Recall*, and the *F*1-score are dimensionless, as they are derived from ratio-based calculations independent of specific measurement units.

Table 2 presents the final metrics for the trained PointNet-based model tested on the test set. The table shows high values for the estimated parameters.

### 3.2. Training the Model Based on PointCloudTransformer

Table 3 presents the parameters set at the beginning of training the PointCloudTransformer neural network. It is worth noting that the parameter k equal to 4 is used to create a graph based on a point cloud using the KNN method [52]. In addition, the StepLR scheduler was used during training, which reduces the learning rate after a certain number of epochs [53]. This enables the model to converge rapidly upon the optimal solution, preventing divergence from the global minimum in subsequent epochs.

**Figure 10:**
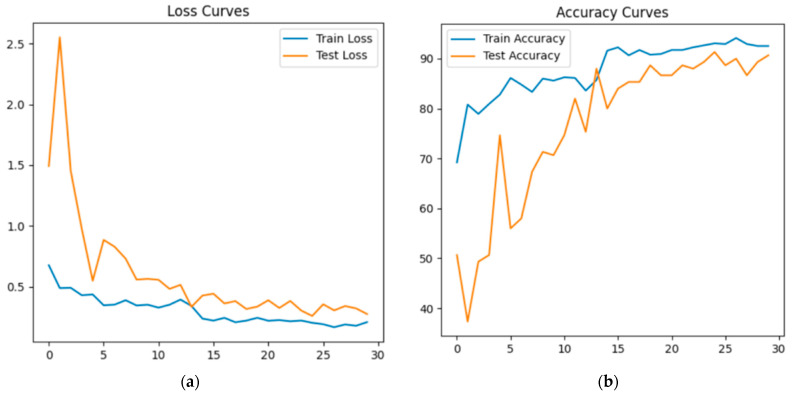
PointCloudTransformer training: (**a**) Loss vs. Epoch graph; (**b**) Accuracy vs. Epoch graph.

Figure 10a shows the training process of the PointCloudTransformer model, and Figure 10b shows the growth of the Accuracy metric during this process.

The graphs show that, at the initial stages, the loss and accuracy curves exhibit significant fluctuations, which is due to the relatively high learning rate. As training progresses, the curves become smoother and flatter, showing that the model has reached an optimal state (solution).

**Figure 11:**
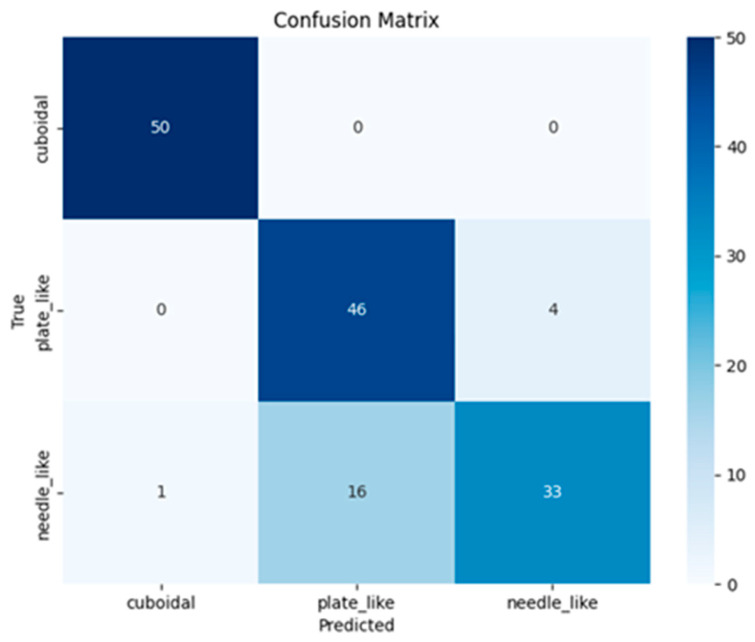
Classification error matrix for PointCloudTransformer.

Figure 11 shows the error matrix obtained during the operation of the trained PointCloudTransformer model on 50 samples from the test set. The result is similar to the previous model.

Table 4 shows the final metric values for the trained PointCloudTransformer model.

As for the PointNet-based model, the best *Precision* value is observed when defining the class “cuboidal”. The class “plate_like” turned out to be the most difficult for the model.

**Figure 12:**
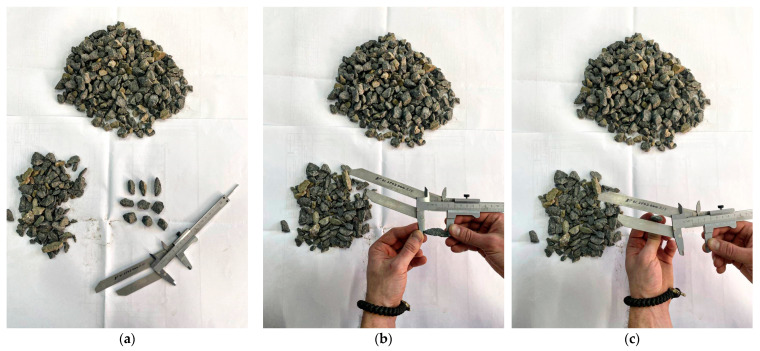
Determination of grain sizes using a movable template (**a**) crushed stone grain sample with a movable template, (**b**) determination of the maximum grain size, and (**c**) determination of the minimum grain size.

To demonstrate the efficiency of the developed intelligent algorithms, an experiment was conducted in laboratory conditions to determine the shape of crushed stone, characterized by the indicator “content of plate-like and needle-like grains”. During the experiment, the time costs for determining the class of crushed stone grains using the proposed algorithms were estimated in comparison with a mobile grain size ratio template. In laboratory conditions, 200 grains were selected, of which 72 grains were cubic, 68 were needle-like, and 60 were plate-like. Measurements with the grain size ratio template were carried out by five specialists with different experience in working with this tool (Figure 12).

To determine the class using intelligent methods, preliminary photo fixation is necessary, which on average takes about 5 s per grain. The total time for collecting 3D images was 16 min 40 s. Pre-processing of the data and its passage through the pre-trained PointNet and PointCloudTransformer models took 20 s and 28 s, respectively (time averaged over 100 measurements).

Table 5 presents the results of the comparison of the indicators for determining the shape of crushed stone grains via the manual method using a moving template and via computer vision methods.

According to the results obtained in Table 5 on 200 crushed stone grains, the accuracy of the proposed computer vision methods is somewhat lower than the accuracy of the manual method using a mobile crushed stone grain size ratio template (86% versus 90%). However, the time spent on determining the shape of crushed stone grains using computer vision methods is 20% less than the manual method using a mobile crushed stone grain size ratio template.

### 3.3. Discussion

Despite the high accuracy rates in determining the classes of crushed stone grains performed by different specialists (90%) compared to the proposed algorithms (86%), it is worth noting the execution time, which is one of the key factors in the field. A specialist’s working time is closely related to their moral and physical indicators at the time of work, while computer vision algorithms are not affected by the fatigue factor. The operating time of the proposed computer vision algorithms is 20% less than the time spent on determining the grain shape using a grain size ratio template. It should be noted that the experiment was conducted on 200 crushed stone grains with sizes of 10–40 mm. Accordingly, when determining a larger number of grains using a moving template and reducing the size of crushed stone grains (5–10 mm), the accuracy will decrease, and the time spent will increase.

It is important to note that the average photo fixation time (5 s) was taken for the calculations, and the models were not launched at the maximum possible capacities in order to check the operating time under limited technical conditions. In the case of using the developed algorithms in real tests when analyzing large volumes of grains, it is possible to achieve a speed of 0.1 s for classifying one grain when using modern GPUs. The photo fixation time can also be reduced to 3–4 s by creating special shooting conditions. In addition, the accuracy of the algorithms will be improved in the course of obtaining additional data arrays and additional training.

Thus, the efficiency and superiority of the proposed computer vision algorithms for determining the shape of crushed stone grains in comparison with the manual method using a template of the ratio of crushed stone grain sizes should be noted, especially with large quantities of controlled large aggregate (more than 100 grains), where the human factor is especially pronounced.

When training neural networks, it is important to estimate the time costs, since the deep learning process is computationally expensive and time-consuming. It is worth noting that training based on transformers uses many more resources (GPU) and takes about 10 times longer.

Comparing the obtained result with the work [41], where 89% accuracy was achieved, it can be noted that the entire mass of crushed stone was used there; the initial data of such a process are easier to obtain and process. The same can be said about the work [43], where limestone was classified by rock type based on visualization using a multi-class support vector machine (SVM). The use of a numerical testing method based on particle flow modeling for testing the bearing capacity coefficient of sorted crushed stone demonstrated high accuracy (the error between the numerical and laboratory results was less than 7%) [44]. However, this method has limitations in terms of the geometric parameters of the samples, which raises some questions and requires detailed clarification due to the variety of the geometric parameters of the crushed stone grains.

The implementation of this study revealed the capacity of intelligent methods to improve the efficiency of building materials control, thereby decreasing costs and mitigating human error in quality control [54,55]. Crushed stone is one of the most popular building materials, which is used in huge quantities as a base for road surfaces and a sub-base for foundations and as the main large filler in the manufacture of concrete composites [56,57,58]. As a rule, the use of cuboid crushed stone is the most desirable. This form of crushed stone ensures the formation of a denser and more stable structure of the base or composite material [59,60]. During the production of crushed stone in plant conditions, special attention is paid to monitoring the content of plate-shaped and needle-shaped grains. The amount of these grains in the total volume of crushed stone produced directly affects its quality and cost. Every crushed stone manufacturer aims to enhance product quality. Currently, most crushed stone production plants control the content of lamellar and needle-shaped grains using classical methods by visual sorting or sifting through slit sieves. These laboratory tests are labor-intensive and time-consuming, which in turn greatly complicates the control of the content of lamellar and needle-shaped grains for each individual batch and grain fraction in real time. In addition, there is a need for the timely adjustment of the operating modes of crushing equipment [61,62]. The intelligent model based on the PointNet and PointCloudTransformer neural networks, specially trained in this study, when implemented in the quality control system at crushed stone plants, will allow for the promp and rapid assessment of impressive volumes of crushed stone for the content of lamellar and needle-shaped grains [63,64,65]. In this study, modifications of PointNet and PointCloudTransformer were used, primarily due to their efficiency at low computational costs (since their implementation in laboratory and field conditions is planned in the future). There are other architectures that show a high classification quality, for example, DGCNN (dynamic graph convolutional neural network). In our study, we did not use DGCNN, despite the assumptions of a high classification accuracy. The main reason was a significant increase in computational costs and implementation complexity, which made this model less suitable for our task of classifying point clouds of crushed stone grains. The limitations associated with the use of computer vision models for classifying crushed stone grains include the following:

→ The need for high-quality 3D photo recording of each sample;
→ Both models work with point clouds, without taking into account the grain texture, which could be useful for analysis;
→ The PointNet-based model loses local features (small-scale structures, irregularities, elevations), analyzing only larger features of the general appearance;
→ The PointCloudTransformer-based model is computationally expensive when processing large data sets and with a large number of neighbors (parameter k) when constructing graphs.

The results will be implemented in lab and field settings in the future. The work will be continued in the direction of searching for additional transformations of the initial data, using other neural networks, and also examining the textural features of materials.

## 4. Conclusions

By implementing two algorithms based on the PointNet and PointCloudTransformer neural networks, specially developed for working with three-dimensional data presented in the form of point clouds and for solving the classification of the shape of crushed stone grains into three classes, the following results were achieved.

(1) An empirical database was created containing information in the form of 3D images of crushed stone grains of three classes.
(2) Parameters were selected for the stable training of intelligent models based on the PointNet and PointCloudTransformer neural networks.
(3) Experimental results showed that the developed models have high accuracy in solving the classification problem: the Accuracy metric = 0.86 for both models.
(4) A comparison of the developed method with the method of manual selection, sieve analysis, or using special equipment was carried out. The comparison showed that the developed approach will reduce manual labor and can also serve as an additional source for verifying the quality of building materials at various stages of construction.
(5) A comparative experiment was conducted on 200 crushed stone grains sized 10–40 mm, demonstrating the efficiency and superiority of the proposed computer vision algorithms over the manual method using a movable template of the crushed stone grain size ratio. With a slightly lower accuracy in determining the shape of crushed stone grains, the computer vision algorithms reduced the time costs by 20% compared to the manual method. It was found that when determining a larger number of grains (more than 200) using a movable template and reducing the size of crushed stone grains (5–10 mm), the accuracy will decrease and the time spent will increase.
(6) The prospect of improving the model lies in the following actions:
  - Application of the developed algorithm to other building materials with similar morphological characteristics;
  - Creation of models capable of taking into account the texture features of the analyzed objects;
  - Classification of the building materials of interest by other visual criteria (color, surface quality);
  - Implementation of the developed models in the process of determining the shape of crushed stone grains in laboratory and field conditions.

In the future, a number of studies are planned on the classification of crushed stone grains using intelligent methods, their verification, and their implementation in laboratory and field conditions. The developed algorithm could, in practice, become part of the process of verifying the quality of building materials at various stages of construction. Its integration with industrial cameras will allow for quick and high-quality analysis of the class affiliation of crushed stone grains, including in real time. The work will be continued in the direction of searching for additional transformations of the original data (for example, translation into 2D), the use of other neural networks (for example, convolutional), and consideration of the textural features of materials.

## Figures and Tables

**Table 1:** PointNet-based model parameters.

Num	Parameter	Value
1	learning rate	0.001
2	weight decay	$10^{-4}$
3	num epochs	50
4	focal loss	α = 0.25, γ = 2
5	batch size	32

**Table 2:** Final metrics for the PointNet-based model.

№	Parameter	*Precision*	*Recall*	*F*1
1	cuboidal	0.95	0.8	0.87
2	plate_like	0.9	0.9	0.9
3	needle_like	0.76	0.88	0.81
4	accuracy	-	-	0.86
5	macro avg	0.87	0.86	0.86
6	weighted avg	0.87	0.86	0.86

**Table 3:** PointCloudTransformer parameters.

Num	Parameter	Value
1	k	4
2	batch_size	8
3	learning rate	0.001
4	weight_decay	$10^{-2}$
5	step_size	15
6	gamma	0.1

**Table 4:** Final metrics for the PointCloudTransformer model.

Num	Parameter	*Precision*	*Recall*	*F*1
1	cuboidal	0.98	1.0	0.99
2	plate_like	0.74	0.92	0.82
3	needle_like	0.89	0.66	0.76
4	accuracy	-	-	0.86
5	macro avg	0.87	0.86	0.86
6	weighted avg	0.87	0.86	0.86

**Table 5:** Results of a comparative experiment to determine the shape of crushed stone grains.

№	Method	Photo Fixation Time, Minutes:Seconds	Operating Time, Minutes:Seconds	Total, Minutes:Seconds	Accuracy, %
1	Grain Size Ratio Template and Visual Method				
1.1	Specialist 1	-	17:36	17:36	98
1.2	Specialist 2	-	18:48	18:48	96
1.3	Specialist 3	-	20:17	20:17	90
1.4	Specialist 4	-	23:41	23:41	84
1.5	Specialist 5	-	26:14	26:14	80
Average results of the method using the grain size ratio template				21:19	90
Computer Vision Algorithms					
2	PointNet	16:40	00:20	17:00	83
3	PointCloudTransformer	16:40	00:28	17:08	88
Average performance of the proposed algorithms				17:04	86

## Data Availability

Data are contained within the article.

## Abbreviations

**Abbreviation**	**Expanded**
AI	Artificial Intelligence
DGCNN	Dynamic Graph Convolutional Neural Network
GPU	Graphics Processing Unit
KNN	K-Nearest Neighbors
QD-LUBE	Quality-Driven Lower Upper Bound Estimation
StepLR	Step Learning Rate
SVM	Support Vector Machine
